# Multifactorial aetiology for non-uremic calciphylaxis: a case report

**DOI:** 10.1080/20009666.2018.1479617

**Published:** 2018-06-12

**Authors:** Sijan Basnet, Niranjan Tachamo, Rashmi Dhital, Biswaraj Tharu

**Affiliations:** a Department of Medicine, Reading Hospital and Medical Center, West Reading, PA, USA; b Maharajgunj Medical Campus, Tribhuvan University, Kathmandu, Nepal

**Keywords:** Calciphylaxis, non-uremic, paraganglioma, sodium thiosulfate, von Kossa stain

## Abstract

Calciphylaxis is commonly associated with end-stage renal disease patients on haemodialysis. We present a rare case of calciphylaxis in a non-uremic patient. The diagnosis was made clinically and confirmed with skin biopsy showing calcification of the dermal and subcutaneous tissues in the von Kossa stain. We believe that the combination of uncontrolled diabetes mellitus, a non-functioning paraganglioma and vitamin D deficiency in a susceptible female patient was responsible for causing calciphylaxis in our patient. An index of suspicion should be maintained by clinicians for calciphylaxis even in patients without uremia.

## 1. Introduction

Calciphylaxis, also known as calcific uremic arteriopathy, is characterized by medial calcification of superficial blood vessels with skin and soft tissue necrosis [1–4]. It affects up to 4% patients on haemodialysis and has a mortality rate of 45–80% with ulcerated lesions [1,5–7]. Of late, it is being reported in patients without end-stage renal disease [8]. It has been associated with primary hyperparathyroidism, malignancy, alcoholic liver disease, proteins C and S deficiencies, connective tissue disorders, vitamin D deficiency, Crohn’s disease, obesity, and medications (calcium, warfarin and corticosteroids) [7–9]. We present a case of a 70-year-old woman diagnosed with non-uremic calciphylaxis.

## 2. Case description

A 71-year-old female presented to the emergency department (ED) with left leg pain, swelling and fever for 1 day. It was preceded by recent unroofing of the nail bed of the great toe. She had poorly controlled type II diabetes mellitus, hypertension and coronary artery disease. In the ED, she was febrile (temperature 103.1°F) with blood pressure of 188/120 mm Hg and pulse of 106/min. Physical examination revealed barely palpable peripheral pulses. The left leg appeared bluish and was extremely tender. White blood cell count on admission was 19,500/µl with neutrophilia. Ultrasound of left lower extremity (LLE) was negative for deep vein thrombosis. She was septic on presentation and was started on vancomycin, cefepime and metronidazole. CT scan of LLE showed diffuse cellulitis/oedema without abscess or osteomyelitis. She underwent LLE arterial duplex which did not show any hemodynamically significant stenosis. The ankle-brachial index showed moderate arterial insufficiency but no evidence of rest pain or critical limb ischemia. On day 3 of presentation, three patches and a bulla appeared on the left leg. Cutaneous vasculitis was suspected. ANA, ANCA, serum C3, C4, Rheumatoid factor, homocysteine, hypercoagulability panel, lipid panel, hepatitis B and hepatitis C were unremarkable. A transesophageal echocardiogram done to rule out septic emboli from infective endocarditis was negative for vegetation. A punch biopsy done from the posteromedial aspect of the left leg showed superficial and deep inflammation with areas of superficial epidermal necrosis and was diagnosed as livedoid vasculitis. Cultures of the skin biopsy were negative. The diagnosis was met with scepticism and the slides were sent to a dermatopathologist. The patient was discharged on wound care and pain control with a review pending.

**Figure 1.**
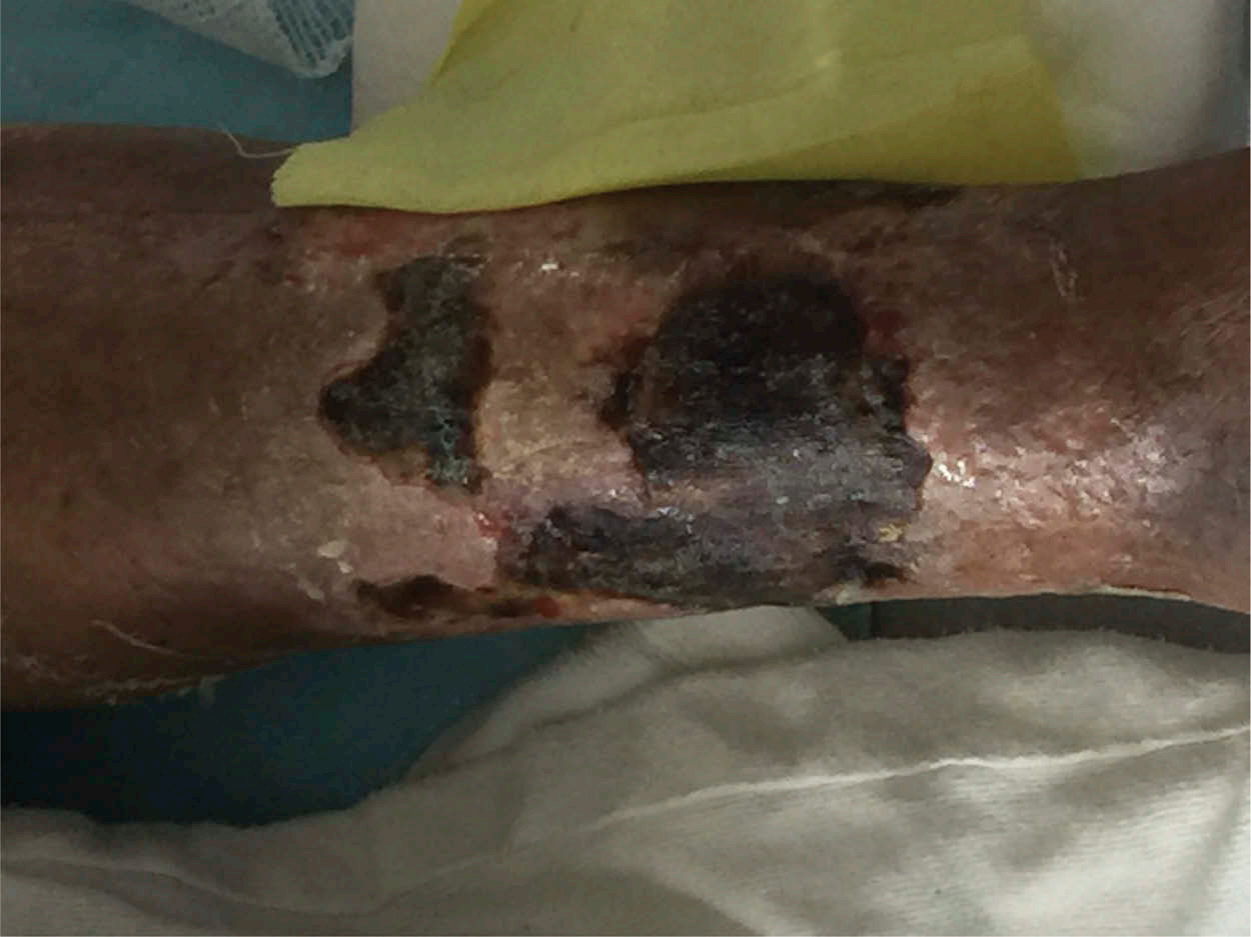
Ulceration and necrosis of the left lower extremity overlying calciphylaxis.

**Figure 2.**
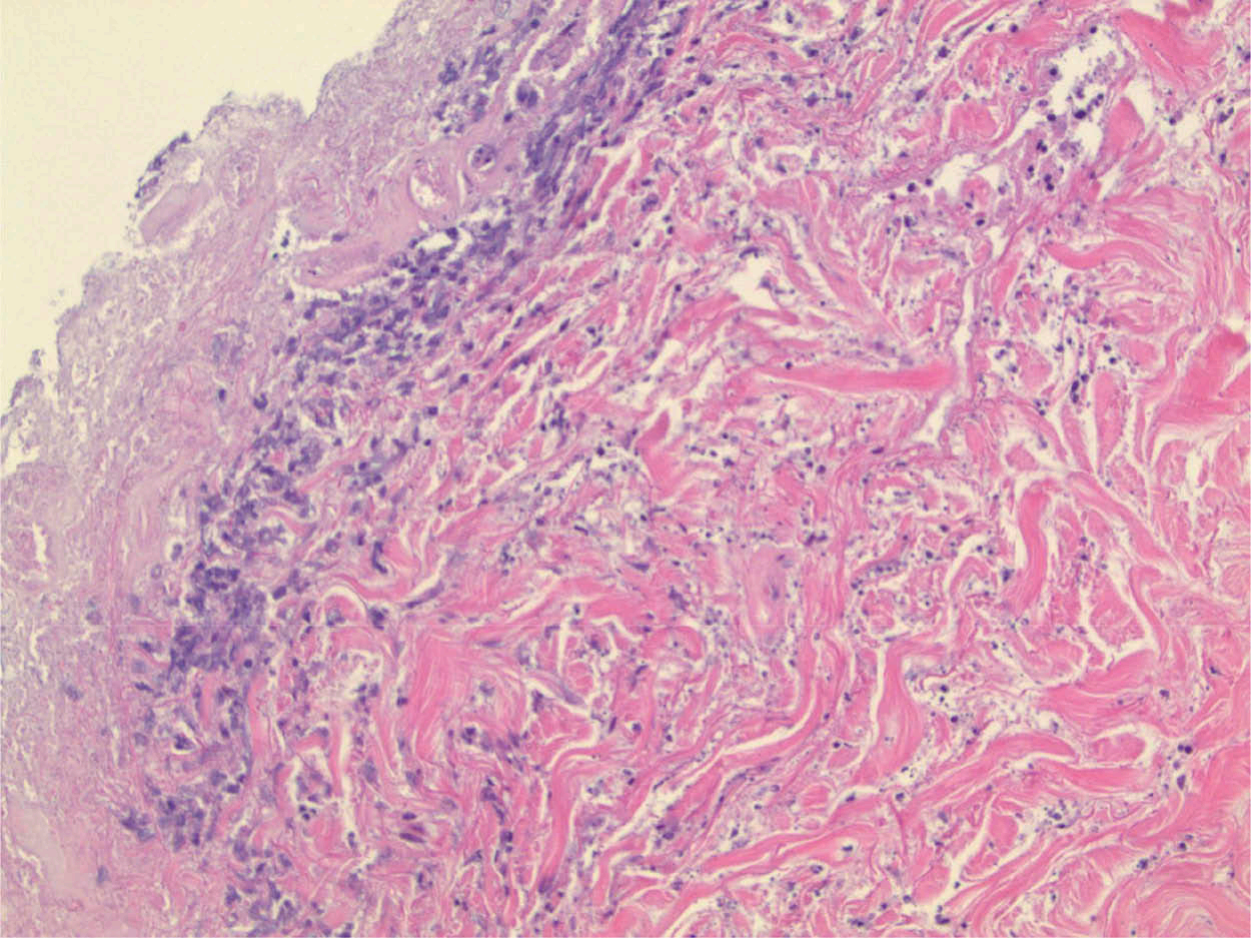
Skin biopsy of necrosed area. Focal areas of lipomembranous, fibrinized vessels and focal basophilic stippling of the subcutaneous vessels.

The patient again presented to the ED a week later with substernal exertional chest pain. Workup for acute coronary syndrome was negative but a significant drop in haemoglobin from 12.3 to 8.4 g/dl was noted since prior admission. The patient underwent upper gastrointestinal endoscopy which showed a sessile 7 mm nodule on the anterior wall of the bulb of the duodenum which was biopsied using cold forceps. Biopsy of the nodule in duodenum showed nests of tumour cells which were positive for chromogranin, synaptophysin, CD56 and pankeratin, consistent with a neuroendocrine tumour. With plasma metanephrines and MIBG whole body scan being normal, there was low suspicion that it was a functional paraganglioma. By this time, the ulcerations were necrotic and had progressed to the left medial leg (Figure 1). Punch biopsies were again taken. Hematoxylin and eosin stained sections reveal an epidermal ulceration, areas of necrosis, neutrophilic inflammatory infiltrate with karyorrhexis in the dermis and subcutaneous tissue. Focal areas of lipomembranous, fibrinized vessels and focal basophilic stippling of the subcutaneous vessels were noted (Figure 2). Paraganglioma as aetiology of calciphylaxis was thought to be unlikely as it was nonfunctional. A von Kossa stain showed calcium deposition in the small vessels of the subcutaneous tissue consistent with a diagnosis of calciphylaxis. A Gram stain, GMS stain, AFB-Fite stain and cultures failed to show an infection.

Workup for an aetiology is detailed in Table 1. Given that the mechanism of her diagnosed calciphylaxis was unclear, sodium thiosulfate was not started. The patient was discharged with recommendations to follow-up with wound care. A year later, the patient has still been following with wound care for poor healing LLE ulcer.

**Table 1.** Potential contributing factors of calciphylaxis.

Serial number	Contributing factors	Workup on our patient
1.	End-stage kidney disease	CKD stage 2 with baseline creatinine: 0.80–1.00 mg/dl and GFR 65–85 ml/min/1.73 sq. m.
2.	Primary hyperparathyroidism	Serum PTH: 48 (reference range: 12–88 pg/ml). Normal calcium, phosphorous with initial vitamin D deficiency.
3.	Malignancy	Diagnosed with duodenal paraganglioma during endoscopy. No history of cholangiocarcinoma, chronic myelocytic leukemia, malignant melanoma, metastatic breast cancer or multiple myeloma.
4.	Alcoholic liver disease	History of occasional alcohol use. CT abdomen showed normal liver. AST: 24 IU/L, ALT: 7 IU/L
6.	Hypoalbuminemia (malnutrition and weight loss)	Serum albumin was 2.8 g/dL on presentation. 2 months prior to presentation was normal (3.7 g/dL)
7.	Connective tissue diseases	Rheumatoid arthritis: Denied joint pain, swelling or morning stiffness. Rheumatoid factor: 11.0 (reference range: ≤14.0 IU/ml) Systemic lupus erythematosus: ANA negative. Didn’t meet criteria for SLE Giant cell arteritis: Denied visual disturbances, headache, jaw claudication and scalp tenderness. ESR 110 (reference range for age: 40 mm/hr) elevated from possible inflammation.
8.	Diabetes mellitus	Poorly controlled type II diabetes mellitus on insulin. HbA1c: 10.2% (reference range: 4.9–6%)
9.	Protein C and S deficiency	No history of chemotherapy. Protein C activity: 94 (reference range: 70–130%) Protein S activity: 56 (reference range: 55–123%)
10.	Crohn’s disease	Normal bowel habits. Colonoscopy a few years ago showed hyperplastic polyp with normal colonic mucosa.
12.	Vitamin D deficiency	History of Vitamin D deficiency. Vitamin D total: 26 (reference range: 25−80 ng/ml) Vitamin D 25 hydroxy: 17.9 (reference range: deficient <20 ng/ml) on initially. 2 weeks later, level was 34.7 on subsequent presentation. Normal calcium, phosphorus, and PTH.
13.	Calcium supplementation	No history of calcium supplementation
14.	Weight loss and obesity	No weight changes; BMI: 26 kg/m^2.^
15.	Warfarin necrosis	No history of warfarin use.
16.	Corticosteroids	No history of steroids use.

## 3. Discussion

With the majority of calciphylaxis seen in the setting of end-stage renal disease, it is believed to result from secondary hyperparathyroidism and elevation of the serum calcium-phosphate product [1]. This does not explain other reported cases of calciphylaxis that lack this association. Thus a multifactorial aetiology is likely [1,10]. End-stage renal disease, secondary hyperparathyroidism, hypercalcemia and hyperphosphatemia are proposed metabolic derangements which result in precipitation of calcium-phosphate in the presence of metallic salts such as iron and aluminium, egg albumin, trauma, etc. [1,9,11,12]. This precipitated product calcify the medium and small vessels and occlude them with resulting ischemia [1,2,9–11]. In our patient, her uncontrolled diabetes mellitus and history of vitamin D deficiency are possible precipitating factors for calciphylaxis. We have low suspicion that her duodenal paraganglioma as it was nonfunctional.

Patients often present with deep dermal pain and palpable masses. The skin lesions are initially violaceous that progress to livedo reticularis and eventually form eschar with non-healing ulcers within days to few weeks [1,6,10]. They have a distal or sacral distribution with a propensity for areas with abundant adipose tissue [2,13].

Calciphylaxis is a clinical diagnosis and does not require skin biopsy for confirmation [11]. Histologically, calciphylaxis features acute and chronic inflammation with calcified blood vessels and necrosis in the dermal and subcutaneous tissues. The classic appearance of the fully evolved disease features a patchy, relatively ‘clean’ necrosis with scant acute and chronic inflammation and a background of calcified small and medium-sized blood vessels in the dermis and subcutis[1]. Calcification is best seen under von Kossa or Alizarin red stains [1,7]. A bone scan can also aid in the detection of calcification in soft tissues [8,10].

Various treatment options, although available, have little benefit [7,10]. Supportive management like pain control and wound care with sodium thiosulfate, hyperbaric oxygen therapy, tissue plasminogen activator, vitamins D and K supplementation, parathyroidectomy, and daily haemodialysis have been described in literature [5,14,15]. Sodium thiosulfate is thought to work by enhancing the solubility of calcium phosphate and removing calcifications [5,10,15]. It also prevents precipitation of calcium deposits [10].

## 4. Conclusion

Calciphylaxis in a non-uremic patient is a rare diagnosis but has a poor prognosis similar to a patient with ESRD [1]. Physicians should be aware of this condition so that early diagnosis and treatment may be instituted.
